# Anti-Inflammatory Effects of Fermented Lotus Root and Linoleic Acid in Lipopolysaccharide-Induced RAW 264.7 Cells

**DOI:** 10.3390/life10110293

**Published:** 2020-11-19

**Authors:** Sung Min Kim, Eun-Jung Park, Jong-Yeon Kim, Jihee Choi, Hae-Jeung Lee

**Affiliations:** 1 Department of Food and Nutrition, College of BioNano Technology, Gachon University, Seongnam, Gyeonggi-do 13120, Korea; arum109ho@gmail.com (S.M.K.); ejpark@gachon.ac.kr (E.-J.P.); rhfemsqpf92@naver.com (J.-Y.K.); mongtaengi@naver.com (J.C.); 2 Institute for Aging and Clinical Nutrition Research, Gachon University, Seongnam, Gyeonggi-do 13120, Korea

**Keywords:** fermented lotus root, anti-inflammatory effect, NF-κB, MAPK

## Abstract

Inflammation is a protective response of the innate immune system. However, aberrant inflammatory responses lead to various diseases. Lotus root, the edible rhizome of **Nelumbo nucifera**, is a popular traditional herbal medicine in East Asia. In a previous study, we reported that fermented lotus root (FLR) alleviated ethanol/HCl-induced gastric ulcers in rats by modulating inflammation-related genes. However, the mechanisms underlying the anti-inflammatory effects of FLR and its major constituent, linoleic acid (LA), are still largely unknown. In this study, we investigated the anti-inflammatory effects of FLR and LA on lipopolysaccharide (LPS)-induced inflammation in RAW 264.7 murine macrophages. We found that FLR inhibited LPS-induced expression of inflammatory mediators through down-regulation of NF-κB activity. Similarly, LA also attenuated LPS-induced inflammatory responses and reduced LPS-induced phosphorylation of proteins associated with NF-κB signaling, such as ERK, JNK, and p38. Overall, our results suggested that FLR and LA may effectively ameliorate inflammatory diseases.

## 1. Introduction

Inflammation is an essential biological response against harmful stimuli including microbial infection and physical injury [1,2]. However, an aberrant inflammatory response can lead to various diseases, such as oral mucositis [3], acute pancreatitis [4], and gastric ulcers [5]. Macrophages recruit other immune cells for resolving inflammation [6], and therefore play an important role in controlling aberrant inflammatory responses. On activation with lipopolysaccharide (LPS), a major component of the outer membrane of gram-negative bacteria, macrophages secrete pro-inflammatory cytokines, such as interleukin-1 beta (IL-1β) and tumor necrosis factor-alpha (TNF-α) [7,8]. LPS is widely used to stimulate macrophages and induce nuclear factor-kappa B (NF-κB), an important regulatory factor of inflammatory responses [9]. Among the transcription factors of the NF-κB family, the NF-κB p50/p65 heterodimer is bound with the inhibitor of kappa B (IκBα) and exists as an inert form in the cytoplasm of unstimulated cells. However, on stimulation by LPS, NF-κB is released and translocated into the nucleus, due to the phosphorylation and degradation of IκBα [10,11].

LPS-induced NF-κB signaling leads to the transcription of inflammation-related enzymes, such as inducible nitric oxide synthase (iNOS) and cyclooxygenase-2 (COX-2) [12,13,14,15]. Similarly, mitogen-activated protein kinases (MAPKs) are also important in regulating the expression of inflammatory mediators [16].

**Nelumbo nucifera**, commonly known as lotus, is cultivated widely in East Asia and used in traditional herbal medicine, food, and beverages. Recent studies have reported that all parts of the lotus plant, flowers, leaves, seeds, and rhizomes, have medicinal value [17,18,19]. For instance, lotus leaves have anti-oxidative [20], anti-viral [21], and anti-obesity properties [22]. Furthermore, lotus seeds have anti-tumor [23] and anti-oxidative effects [19,24], while the embryos of seeds are used as a sedative and antipyretic [25]. Moreover, accumulated experimental evidences demonstrate that the lotus root (the edible rhizome of *N. nucifera*) has many beneficial effects including anti-oxidant activity [26], hypoglycemic effects [27], prevention of non-alcoholic fatty liver disease [18], alleviating hepatic steatosis [28], immune-enhancing effects [29], and anti-inflammatory activity [30]. In the food processing industry, fermentation by microorganisms is widely used to enhance the nutritional value as well as organoleptic quality of food, including flavor and aroma [31]. Recent studies have reported that biological effects of plant-based foods, such as antioxidant or anti-obesity effects were enhanced through fermentation [32,33].

In an earlier study, we demonstrated the anti-inflammatory effects of fermented lotus root (FLR) on ethanol/HCl-induced gastric ulcers in rats [34], suggesting that FLR can be used as a functional food for attenuating inflammatory diseases. Notably, one the major constituents of *N. nucifera*, linoleic acid (LA), is a polyunsaturated ω-6 essential fatty acid, which humans obtain from foods or supplements. However, the role of LA in inflammation remains controversial [35]. To the best of our knowledge, the mechanisms underlying the anti-inflammatory effects of FLR and LA have not yet been elucidated. Therefore, in this study, we investigated the mechanisms underlying the anti-inflammatory effects of FLR and LA in LPS-induced RAW 264.7 murine macrophages.

## 2. Materials and Methods 

### 2.1. Preparation of FLR and LA

Fermented lotus root (FLR) was obtained in powder form from Hwashin Farming Corporation (Gyeongsangnam-do, Korea). The method used for the lotus root fermentation has been detailed in our previous study [34]. The FLR powder was dissolved in dimethyl sulfoxide (DMSO) and was then filtered with 0.20 μM syringe filter as a stock solution. Linoleic acid (LA, ≥99%; 9-cis, 12-cis-18:2) was purchased from Sigma-Aldrich (Saint Louis, MO, USA) and dissolved in absolute ethanol. The stock solution was stored at −20 °C.

### 2.2. LA Detection by Gas Chromatography with Flame Ionization Detector (GC-FID)

LA in FLR was detected by GC-FID using an Agilent 7890 GC system (Agilent Technologies, Inc., Santa Clara, CA, USA). The conditions of analysis were as follows: the column (HP-FFAP, 30 m × 0.25 mm × 0.25 μm, Agilent Technologies) was used to separate sample. The carrier gas was nitrogen and set at a flow rate of 1 mL/min. The injection temperature was 230 °C and detector temperature was 250 °C; the injection volume was 1 μL with 1:50 split ratio. The initial oven temperature was 120 °C (hold time 2 min) and raised at 4 °C/min to 240 °C (hold time 20 min). 

### 2.3. Cell Culture

Murine macrophage RAW 264.7 cells were obtained from American Type Culture Collection (Manassas, VA, USA) and cultured in Dulbecco’s modified Eagle’s medium supplemented with 10% fetal bovine serum, penicillin (100 units/mL), and streptomycin (100 µg/mL). All cell culture reagents were purchased from GIBCO (Gaithersburg, MD, USA). Cells were maintained at 37 °C, in an incubator with a humidified atmosphere of 95% air and 5% CO_2_.

### 2.4. Cell Viability Assay

Cell viability assay was carried out using a CCK-8 (Dojindo Laboratories, Kumamoto, Japan) according to the manufacturer’s protocol. RAW 264.7 cells were plated in 96-well plate at a concentration of $1\times 10^{4}$ cells/well. After 24 h, the cells were treated with FLR (25, 50, 100, and 200 μg/mL) or LA (1, 10, and 100 μM) with or without 1 μg/mL lipopolysaccharide (LPS; from Escherichia coli O26:B6; Sigma-Aldrich, Saint Louis, MO, USA), and incubated for another 24 h. Finally, 10 μL of the CCK-8 solution was added to each well and incubated at 37 °C for 2 h in the dark. The absorbance was determined at 450 nm using an Epoch Microplate Spectrophotometer (BioTek Instruments, Inc., Winooski, VT, USA). The cell viability was calculated according to the following formula, and expressed as a percentage of the VC.

(1) $$\mathrm{Cell}\;\mathrm{viability}\;\left(\%\right)=\;\frac{\mathrm{the}\;\mathrm{absorbance}\;\mathrm{of}\;\mathrm{the}\;\mathrm{treated}\;\mathrm{group}}{\mathrm{the}\;\mathrm{absorbance}\;\mathrm{of}\;\mathrm{the}\;\mathrm{vehicle}\;\mathrm{control}\;\mathrm{group}}\;\times \;100$$

### 2.5. Nitric Oxide (NO) Production

Cells were plated in a 6-well plate at a concentration of $4\times 10^{5}$ cells/well. After 24 h, cells were co-treated with LPS and different concentrations of either FLR or LA. The culture medium was collected after 24 h, and NO production in the culture medium was measured using Griess Reagent System (Promega, Madison, WI, USA) based on the Griess reaction. The absorbance was determined at 520 nm.

### 2.6. RNA Isolation and cDNA Synthesis

Cells treated with LPS and FLR or LA were harvested, and total RNA from cells was extracted and purified using Total RNA Extraction Kit (iNtRON Biotechnology, Gyeonggi-do, Korea) according to the manufacturer’s instructions. Purity and concentration of extracted RNA were quantified by measuring the absorbance ratio at 260/280 nm using a Take3 Micro-Volume Plate (BioTek Instruments, Inc.). First-strand complementary DNA (cDNA) was synthesized using GoScript™ Reverse Transcriptase (Promega, Madison, WI, USA) according to the manufacturer’s instructions.

### 2.7. Immune Gene Expression Analysis by Quantitative Reverse Transcription PCR (qRT-PCR)

Immune gene expressions of RAW 264.7 including *Nos2*, *Ptgs2*, *Tnf-α, Il1b, and Il6* were quantified using *Actb* as a housekeeping gene by QuantStudio 3 Real-Time PCR Instrument (Thermo Fisher Scientific, Waltham, MA, USA). Total volume of real-time RT-PCR reaction mixture was 20 μL with a 96-well format, included 5.5 μL cDNA template, 2 μL primer mixture (10 μM, 1 μL forward and reverse primer each), 2.5 μL DEPC-treated water, 10 μL TB Green^®^ Premix Ex Taq™ II (Takara Bio Inc., Kusatsu, Shiga, Japan). All primers were obtained from Macrogen (Seoul, Korea), and sequences were referenced from Chaiwat Monmai et al. [36]. The primer sequences used are shown in Table 1. 

### 2.8. Luciferase Assay

The details of the luciferase assay were described in our previous study [37]. In brief, cells were transfected with NF-κB promoter for 24 h, and then incubated with LPS (1 μg/mL) and either FLR (25 to 200 μg/mL) or LA (1 to 100 μM) for another 24 h. After incubation, the culture medium was replaced with Nano-Glo^®^ Dual-Luciferase^®^ Reporter Assay System (Promega, Madison, WI, USA) according to the manufacturer’s instructions. Luciferase activity was determined using a GloMax^®^ Discover Microplate Reader (Promega, Madison, WI, USA).

### 2.9. Western Blot Analysis

The effects of FLR and LA on NF-κB and MAPK signaling in LPS-induced RAW 264.7 cells were examined by western blot analysis. The details of the western blot analysis have been described in our previous study [37]. Antibodies against NF-κB p65, phospho-NF-κB p65 (Ser536), IκBα, phospho-IκBα (Ser32), ERK, phospho-ERK (Thr202/Tyr204), p38, phospho-p38 (Thr180/Tyr182), JNK, phospho-JNK (Thr183/Tyr185) were obtained from Cell Signaling Technology (Danvers, MA, USA), and β-actin was obtained from Abcam (Cambridge, MA, USA). Horseradish peroxidase (HRP)-conjugated secondary antibodies were purchased from Promega (Madison, WI, USA). 

### 2.10. Immunofluorescence Staining

Cells cultured on glass coverslips in 6 well plate and treated with LPS and FLR or LA were fixed with 3.7% para-formaldehyde for 10 min. Next, cells were washed three times with phosphate-buffered saline containing 0.1% Triton X-100 (Sigma-Aldrich, Saint Louis, MO, USA) for 10 min, each time. Cells were then permeabilized with 0.5% Triton X-100 for 5 min. Following permeabilization, cells were washed three times and blocked with 5% Bovine Serum Albumin (Sigma-Aldrich, Saint Louis, MO, USA) for 1 h. Cells were incubated with a NF-κB p65 antibody (1:400, Cell Signaling Technology) for 1 h. After washing three times again, cells were incubated with Alexa 555-conjugated anti-rabbit IgG secondary antibody (Thermo Fisher Scientific, Waltham, MA, USA) for 1 h under the dark conditions. Nuclei were stained with 1 μg/mL of 4′,6-Diamidino-2-phenylindole dihydrochloride (DAPI, Sigma-Aldrich, Saint Louis, MO, USA). Finally, after mounting the coverslips, immunofluorescence was observed using a fluorescence microscope (Korea Lab Tech., Gyeonggi-do, Korea).

### 2.11. Statistical Analysis

The experiments were carried out in triplicates, and statistical data from experiments were obtained using GraphPad Prism 5 software (GraphPad Software, La Jolla, CA, USA) one-way analysis of variance (ANOVA) followed by Tukey’s post hoc test. Data were expressed as the mean ± standard error of the mean (SEM). * *p* < 0.05, ** *p* < 0.01, *** *p* < 0.001 represent comparisons between the LPS only-treated group versus the treated group. 

## 3. Results

### 3.1. Effects of FLR on Cell Viability

**Figure 1:**
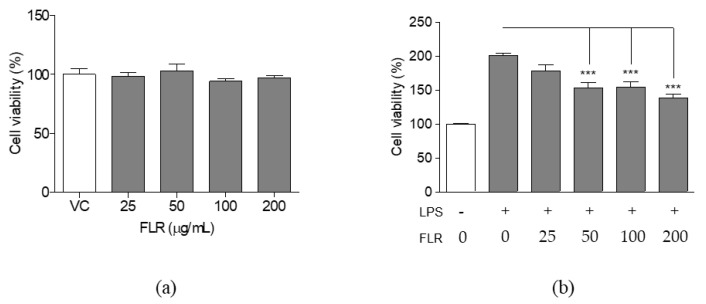
Effects of fermented lotus root (FLR) on cell viability. Cell viability in (**a**) RAW 264.7 murine macrophages treated with 25 to 200 μg/mL FLR for 24 h. (**b**) RAW 264.7 cells co-treated with FLR and lipopolysaccharide (LPS, 1 μg/mL) for 24 h. Cell viability is expressed as a percentage of the vehicle control (VC). The data represent mean ± SEM, *n* = 3. *** *p* < 0.001 compared to LPS-only treated group.

Cell viability was measured using the cell counting kit-8 (CCK-8) assay, following exposure to different concentrations of FLR, to investigate the cytotoxic effects of FLR on RAW 264.7 cells. As shown in Figure 1a, cell viability of unstimulated cells was not significantly affected at FLR concentrations of 25, 50, 100, and 200 μg/mL. In contrast, proliferation in LPS-stimulated group significantly reduced in a dose-dependent manner in the presence of FLR (Figure 1b). Therefore, the FLR concentration of 200 μg/mL was selected as the highest experimental concentration for this study, as no significant cytotoxicity was observed for any of the tested concentrations.

### 3.2. Effects of FLR on NO Production and Expression of Pro-Inflammatory Mediators 

**Figure 2:**
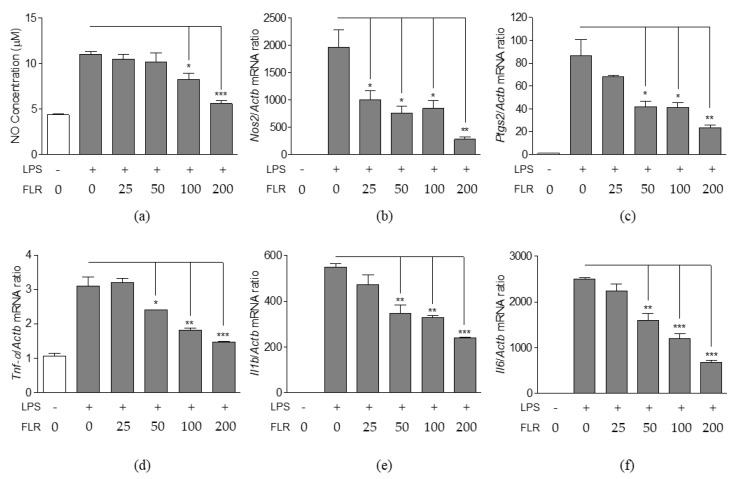
Effects of FLR on nitric oxide (NO) production and immune gene expression. RAW 264.7 cells were co-treated with LPS (1 μg/mL) and FLR (25 to 200 μg/mL) for 24 h. (**a**) NO production in culture medium (**b**–**f**) mRNA expression of (**b**) *Nos2*, (**c**) *Ptgs2*, (**d**) *Tnf-α*, (**e**) *Il1b*, and (**f**) *Il6* quantified by RT-PCR. The data represent mean ± SEM, *n* = 3. * *p* < 0.05, ** *p* < 0.01, *** *p* < 0.001 compared to LPS-only treated group.3.3. Effects of FLR on the activation and nuclear translocation of NF-κB p65.

Nitric oxide (NO) is an important molecular effector in the inflammatory response [38]. NO is also produced by LPS-stimulated macrophages, along with other pro-inflammatory mediators. We analyzed the effects of FLR on NO production and the expression of pro-inflammatory mediators in RAW 264.7 cells. As shown in Figure 2a, FLR suppressed LPS-induced NO production in RAW 264.7 cells. However, the decrease was significant only for higher concentrations of FLR (100, 200 μg/mL). At the highest concentration of FLR treatment, a two-fold decrease in LPS-induced NO production was observed as compared to the LPS-only treated group. Further, LPS induced the expression of inducible nitric oxide synthase (*Nos2*), prostaglandin-endoperoxide synthase 2 (*Ptgs2*), tumor necrosis factor-alpha (*Tnf-α*), interleukin-1 beta (*Il1b*), and interleukin-6 (*Il6*) genes. In contrast, FLR treatment significantly suppressed the LPS-induced expression of these genes in a dose-dependent manner (Figure 2b–f).

**Figure 3:**
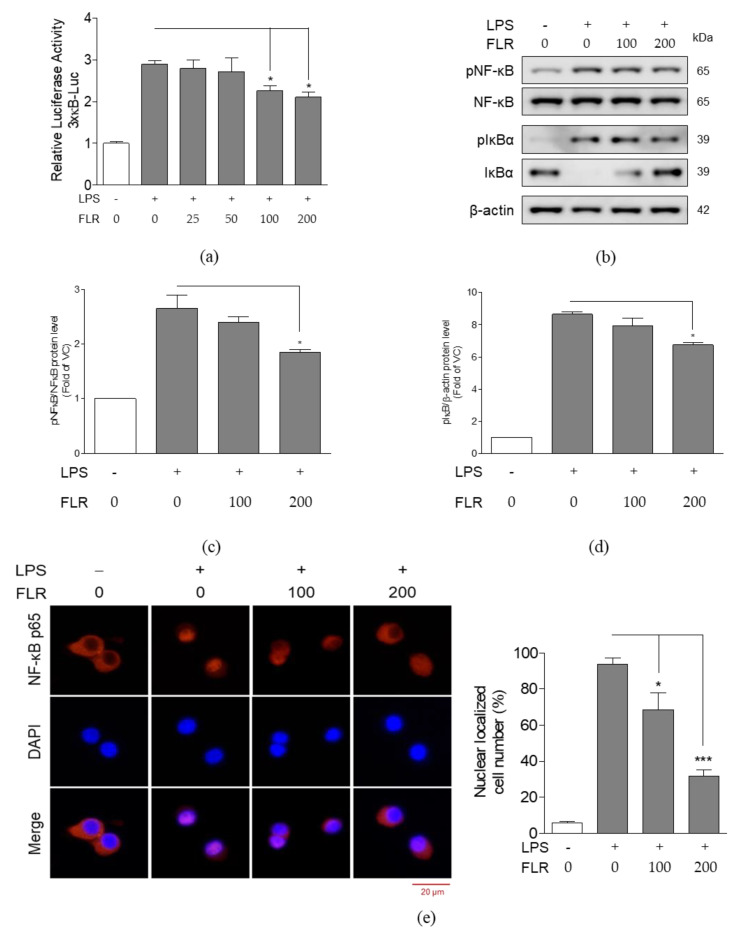
Effects of FLR on NF-κB signaling. (**a**) NF-κB promoter activity in transfected RAW 264.7 cells co-treated with LPS (1 µg/mL) and FLR (25 to 200 μg/mL) for 24 h. (**b**–**d**) Western blot analysis of phosphorylated and unphosphorylated NF-κB and IκBα extracted from cells co-treated with 1 μg/mL LPS and 100, 200 μg/mL FLR for 1 h. The data represent mean ± SEM, *n* = 3. * *p* < 0.05, *** *p* < 0.001 compared to LPS-only treated group. (**e**) Representative images from immunofluorescence. The nucleus (blue color) was stained with 4′,6-Diamidino-2-phenylindole dihydrochloride (DAPI) and NF-κB p65 (red color) was stained with appropriate antibodies. Images were captured at 400× magnification. The scale bar indicates 20 μm. The scale bar indicates 20 μm. The number of cells with p65 nuclear translocation were counted for the statistical analysis in three random fields of each group.

Inflammatory signaling is initiated by the phosphorylation and degradation of inhibitor of kappa B (IκBα), and subsequent release and translocation of nuclear factor kappa B (NF-κB) p65 from the cytoplasm into the nucleus. Therefore, we analyzed the inhibitory effects of FLR on NF-κB activation. We found that LPS increased NF-κB promoter activity in transfected RAW 264.7 cells whereas FLR treatment (100 and 200 μg/mL) significantly reduced the luciferase activity induced by LPS (Figure 3a). Further, western blot analysis confirmed that LPS increases the degradation of IκBα and phosphorylation of NF-κB p65 and IκBα and FLR treatment blocks the LPS-induced degradation of IκBα and phosphorylation of NF-κB p65 and IκBα (Figure 3b–d). 

Furthermore, we analyzed the translocation of NF-κB p65 to the nucleus using immunofluorescence (Figure 3e). The translocation of NF-κB p65 from the cytoplasm to the nucleus was evident in LPS-only treated group as compared to the vehicle control (VC) group. However, nuclear translocation of NF-κB p65 was partially inhibited in the FLR-treated group. These results were in agreement with the FLR suppression of inflammatory mediators (Figure 2) and further suggested that FLR inhibits the activation of NF-κB in RAW 264.7 cells.

### 3.3. Detection of LA in FLR and Effects of LA on Cell Viability

**Figure 4:**
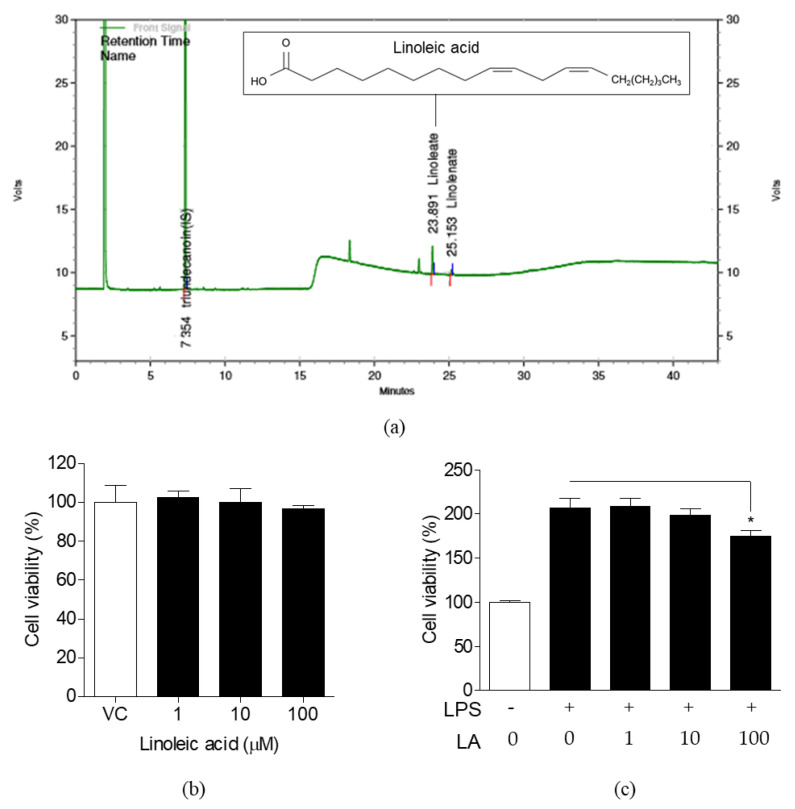
Chromatogram of linoleic acid (LA) and effects of LA on cell viability. (**a**) Chromatogram of LA was obtained using gas chromatography with flame ionization detector (GC-FID). (**b**) Cell viability in RAW 264.7 cells treated with different concentrations of LA (1, 10, and 100 μM) for 24 h. (**c**) Cell viability in RAW 264.7 cells co-treated with LPS (1 μg/mL) and LA for 24 h. Cell viability was measured by CCK-8 assay and expressed as a percentage compared to that of VC. The data represent mean ± SEM, *n* = 3, * *p* < 0.05 compared to LPS-only treated group.

In our previous study, we found that FLR contained LA [34] and the chromatogram of LA obtained by gas chromatography with flame ionization detector (GC-FID) is shown in Figure 4a. The amount of LA, deduced according to the formula offered by Ministry of Food and Drug Safety (Korea), was around 153 mg/100 g FLR. As shown in Figure 4b, significant cytotoxic effects were not observed in normal RAW 264.7 cells treated with LA at concentrations of 1, 10, and 100 μM. However, cell proliferation induced by LPS was significantly reduced in the 100 μM LA-treated group (Figure 4c). As lower concentrations of LA (1 to 100 μM) did not show cytotoxicity in both, the unstimulated as well as LPS-stimulated groups, we decided to carry out further analysis at concentrations of 1, 10, and 100 μM LA.

### 3.4. Effects of LA on NO Production and Expression of Pro-Inflammatory Mediators

**Figure 5:**
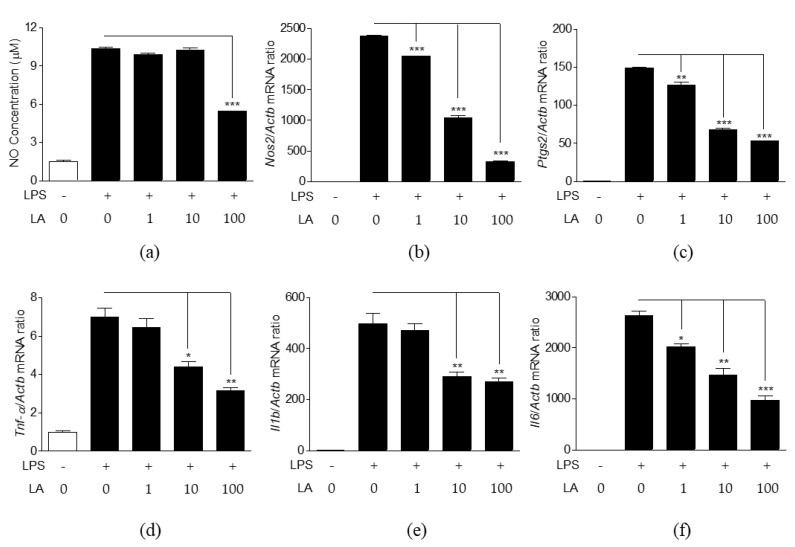
Effects of LA on NO production and expression of immune genes. Cells were co-treated with LPS (1 μg/mL) and LA at the concentrations of 1, 10, and 100 μM for 24 h. (**a**) NO production in culture medium (**b**–**f**) mRNA levels of immune genes *Nos2(b), Ptgs2 (c)*, *Tnf-α (d)*, *Il1b* (e), and *Il6 (f)*. The data represent mean ± SEM, *n* = 3. * *p* < 0.05, ** *p* < 0.01, *** *p* < 0.001 compared to LPS-only treated group.

The effects of LA on NO production and pro-inflammatory mediator expression were analyzed in LPS-induced cells (Figure 5). Lower concentrations of LA (1, 10 μM) were not effective in reducing NO production, whereas treatment with 100 μM LA reduced NO production significantly (Figure 5a). However, LA effectively decreased the expression of *Nos2*, *Ptgs2*, *Tnf-α*, *Il1b*, and *Il6* genes at all concentrations in a dose-dependent manner (Figure 5b–f).

### 3.5. Effects of LA on the Activation and Translocation of NF-κB p65 

**Figure 6:**
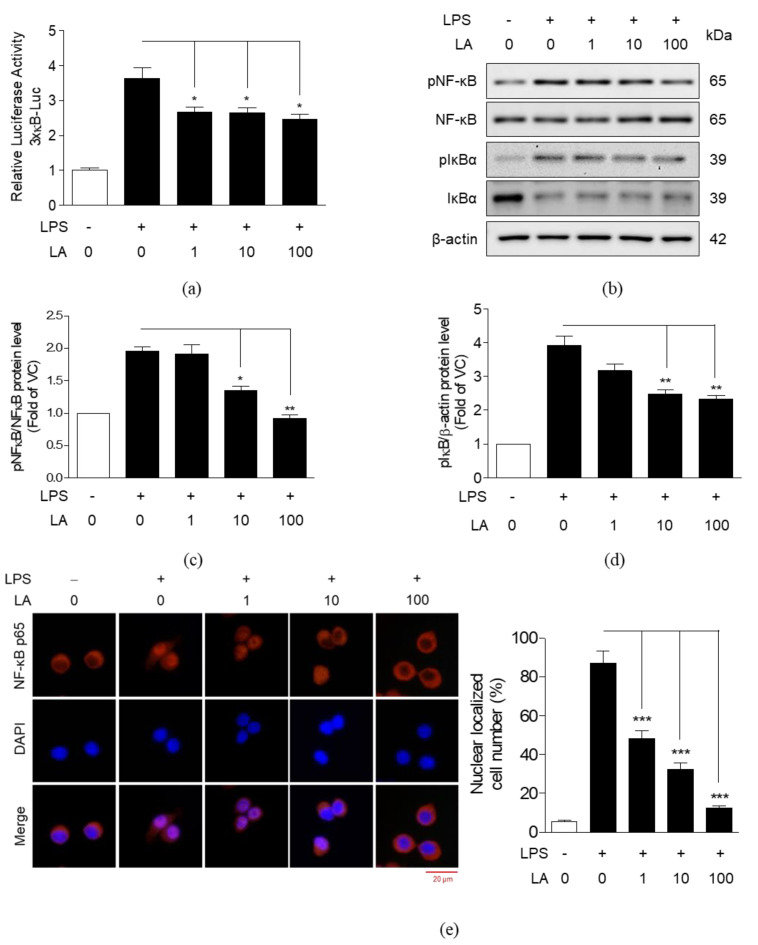
Effects of LA on NF-κB signaling. (**a**) NF-κB promoter activity in transfected RAW 264.7 cells co-treated with LPS and LA (1 to 100 μM) for 24 h. (**b**–**d**) Western blot analysis of phosphorylated and unphosphorylated NF-κB and IκBα proteins extracted from cells co-treated with LPS (1 μg/mL) and LA (1 to 100 μM) for 1 h. The data represent mean ± SEM, *n* = 3. * *p* < 0.05, ** *p* < 0.01, *** *p* < 0.001 compared to LPS-only treated group. (**e**) Representative images from immunofluorescence. The nucleus (blue color) was stained with 4′,6-Diamidino-2-phenylindole dihydrochloride (DAPI) and NF-κB p65 (red color) was stained with appropriate antibodies. Images were captured at 400× magnification. The scale bar indicates 20 μm. The number of cells with p65 nuclear translocation were counted for the statistical analysis in three random fields of each group.

The effects of LA on activation and nuclear translocation of NF-κB p65 were investigated in the same manner as those of FLR were investigated. We observed that LA treatment (1 to 100 μM) decreased LPS-induced luciferase activity in transfected cells, as shown in Figure 6a. Protein expression was visualized by western blot analysis. LPS-induced increase in phosphorylation of NF-κB p65 and IκBα was reduced, and degradation of IκBα was blocked when cells were treated with LA (Figure 6b–d). Nuclear translocation of NF-κB p65 was also observed by immunofluorescence (Figure 6e). The region stained with NF-κB p65 antibody (red color) was consistent with the region stained with 4′,6-Diamidino-2-phenylindole dihydrochloride (DAPI, blue color), indicating the translocation of NF-κB p65 from cytoplasm to the nucleus in LPS-induced group. In contrast, NF-κB p65 was not translocated from cytoplasm into the nucleus in LA-treated cells. These results were similar to the FLR-treated cells and suggested that LA inhibits NF-κB activation in LPS-induced RAW 264.7 cells.

### 3.6. Effects of LA on MAPK Activation 

**Figure 7:**
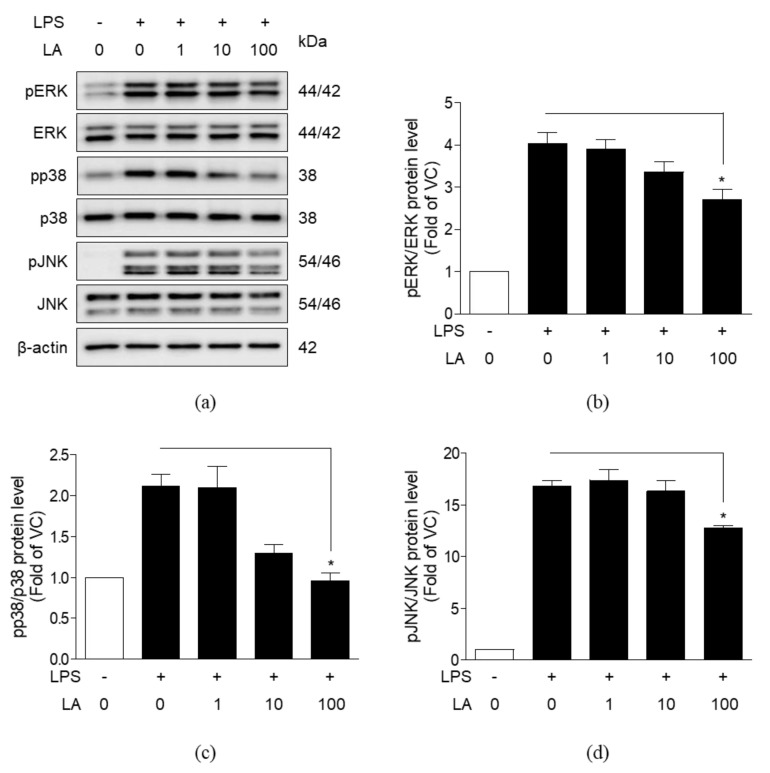
Effects of LA on phosphorylation of extracellular signal-regulated kinase (ERK), p38, and c-Jun N-terminal kinase (JNK). RAW 264.7 cells were co-treated with LPS (1 μg/mL) and 1 to 100 μM LA for 1 h. Western blot analysis of phosphorylated and unphosphorylated ERK, p38, and JNK proteins to evaluate MAPK activation The images above are representatives of triplicate experiments. The data represent mean ± SEM, *n* = 3. * *p* < 0.05, compared to LPS-only treated group.

To evaluate whether LA affects mitogen-activated protein kinase (MAPK) signaling, we analyzed the effects of LA on the phosphorylation of extracellular signal-regulated kinases (ERK), p38, and c-Jun N-terminal kinase (JNK) in LPS-induced RAW 264.7 cells by western blot analysis. LPS increased the phosphorylation of ERK, p38, and JNK (Figure 7). Treatment with 1 and 10 μM LA had no effect on phosphorylation, whereas treatment with 100 μM LA decreased the phosphorylation of ERK, p38, and JNK proteins.

## 4. Discussion and Conclusions

Inflammation is an essential immune response to protect our body against harmful stimuli, such as infection, stress, and physical injury. As the inflammatory response is a defense mechanism of our body against tissue injury, the lack of a normal inflammatory response could lead to tissue damage. An acute inflammation phase initiates the inflammatory process, wherein immune cells are rapidly recruited to the injury site. However, a persistent acute inflammatory response may cause severe pain and develop into various acute inflammatory diseases including gastric ulcer, acute hepatitis, and autoimmune disorders [39,40,41]. 

The inflammatory response is accompanied by the secretion of NO and pro-inflammatory cytokines by immune cells. These are important inflammatory mediators involved in immune response processes, such as extracellular signaling and vasodilation. However, a high amount of NO, due to continuous activation of iNOS, could cause toxic effects, such as oxidative stress and aggravation of inflammation [38,42]. Cytokines released from immune cells also play a critical role in inflammation. Pro-inflammatory cytokines, including TNF-α, IL-1β, and IL-6, which are rapidly produced in the early stage of inflammation, could be harmful when overproduced, similar to NO [8,43]. We observed that FLR and LA reduce the expression of *Nos2*, *Ptgs2*, *Tnf-α*, *Il1b*, and *Il6* genes as well as production of NO in LPS-induced RAW 264.7 cells, thus suggesting that FLR and LA may potentially regulate production of NO and cytokines in inflammatory responses. A total of 10 μM of LA reduced mRNA expression of *Nos2* and *Ptgs2* comparable to 10 μM, of the NF-κB inhibitor PDTC (Figure 5 and Figure S1).

MAPK proteins are important in inflammation as well as cell proliferation and differentiation. MAPK proteins, such as ERK, p38, and JNK are activated by extracellular stimuli at the cell membrane. Phosphorylated ERK and p38 induce the transcriptional activity of NF-κB, and ultimately release inflammatory mediators, including NO, TNF-α, and IL-6 [44,45,46]. JNK is also involved in inflammatory response and phosphorylates c-Jun, which in turn induces the expression of the activator protein 1 transcription factor [47]. Besides, it is reported that JNK induces the degradation of IκBα [48].

NF-κB is one of the most important transcriptional factors involved in the inflammatory response. NF-κB is generally localized in the cytoplasm in an inactive form, as a heterodimer with IκB. Phosphorylation of IκB is essential for the release of NF-κB. When IκB kinase is activated by various extracellular signals, IκB is phosphorylated and detaches from NF-κB. Dissociated IκB is ultimately degraded following ubiquitination, whereas the liberated NF-κB is translocated from cytoplasm into the nucleus. In the nucleus, NF-κB binds to a specific site on the target gene and induces target gene expression. Thus, phosphorylation of NF-κB and IκB leads to the transcription of target gene such as iNOS, COX-2, and cytokines [49,50]. Therefore, excessive activation of NF-κB may result in an enhanced inflammatory response. 

In this study, immunofluorescence evidence confirmed translocation of NF-κB p65 into the nucleus by LPS in contrast to the cytoplasmic localization of NF-κB p65 in unstimulated cells. However, treatment with FLR and LA apparently blocked the nuclear translocation of NF-κB p65. Thus, our results suggest that FLR and LA suppress the activation of NF-κB by blocking the phosphorylation of NF-κB and IκBα. 

Both MAPK and NF-κB signaling are triggered when Toll-like receptor 4 (TLR4) recognize and bind LPS in macrophages. Phosphorylated MAPK and NF-κB induce transcription of inflammatory mediators, and thereby aggravate inflammation [51]. Therefore, MAPK/NF-κB signaling should be controlled against excessive activation. Recent accumulated evidences have demonstrated that inflammation is suppressed by the inhibition of MAPK/NF-κB signaling in LPS-induced macrophage cell lineage [52,53]. 

The role of dietary LA on inflammation remains controversial. Several in vitro and in vivo studies have demonstrated anti-inflammatory effects of LA [54,55]. LA reduced inflammatory responses in microglial cells [54] and decreased IL-1β, IL-6, and NF-κB activation in the wound healing rat model [55]. On the contrary, it has been reported that LA might accelerate inflammation. High levels of LA intake induced pro-inflammatory markers such as TNF-α, IL-1β, and IL-6 in rats [56] and elevated the risk of ulcerative colitis in a nested case–control study [57]. These effects have been attributed to arachidonic acid, which is synthesized from LA and produces pro-inflammatory mediators [58,59]. Despite this, dietary LA has not exhibited any portent of inflammation in mice models [60] and a high dietary ω-6 fatty acid did not evidently affect inflammation when it was provided instead of saturated fatty acids [61]. Furthermore, the tissue arachidonic acid content was not correlated with the increase of dietary LA [62]. 

In this study, LA decreased pro-inflammatory markers in RAW 264.7 cells. These results are similar to a previous report in which mRNA expression of *Nos2*, *Tnf-α, Il1b,* and *Il6* induced by LPS in macrophages was decreased in the presence of 150 μM LA isolated from *Agaricus brasiliensis* [63]. Moreover, we observed that LA exhibited anti-inflammatory effects through the suppression of MAPK/NF-κB signaling and decreased phosphorylation of NF-κB, IκBα, ERK, p38, and JNK. Additionally, LA not only blocked nuclear translocation of NF-κB p65 but also prevented transcriptional activity of NF-κB. Overall, our results strongly support the anti-inflammatory effects of LA. Future studies with positive control are needed to confirm the results. 

Our ultimate goal is to evaluate the potential of FLR as a novel health functional food which has anti-inflammatory effect. A total of 100 g of FLR powder contains 153 mg of linoleic acid. The amount of LA contained in the highest concentration, 200 μg/mL FLR, can be converted to around 1.08 μM. Additionally, in our previous study, it was found that the FLR powder 100 g contained 301 mg of total flavonoid, 1110 mg of total polyphenol. Although the amount of LA contained in FLR is not large, LA, which has not been studied much on anti-inflammatory effect compared to polyphenols or flavonoids, was used in the experiment. In addition, if FLR will be developed as a novel health functional food (as powder/tablet form), it is thought that we can intake not only linoleic acid but also a large amount of active ingredients and get anti-inflammatory effects.

Taken together, the results of this study provide mechanistic support to our previous study where we showed that gastroprotective effects of FLR in vivo [34]. To the best of our knowledge, this study is the first demonstration of the anti-inflammatory effects of FLR and its component LA in LPS-induced macrophages. Further clinical studies are needed to confirm the anti-inflammatory effects of FLR demonstrated in this study.

## Figures and Tables

**Table 1:** Primer sequences used for RT-PCR analysis.

Genes	Forward Primer (5′–3′)	Reverse Primer (5′–3′)	Product Size (bp)
*Actb*	CCACAGCTGAGAGGAAATC	AAGGAAGGCTGGAAAAGAGC	193
*Nos2*	TTCCAGAATCCCTGGACAAG	TGGTCAAACTCTTGGGGTTC	180
*Ptgs2*	AGAAGGAAATGGCTGCAGAA	GCTCGGCTTCCAGTATTGAG	194
*Tnf-* *α*	ATGAGCACAGAAAGCATGATC	TACAGGCTTGTCACTCGAATT	276
*Il1b*	GGGCCTCAAAGGAAAGAA	TACCAGTTGGGGAACTCTGC	183
*Il6*	AGTTGCCTTCTTGGGACTGA	CAGAATTGCCATTGCACAAC	191

## Supplementary Materials

The following are available online at https://www.mdpi.com/2075-1729/10/11/293/s1, Figure S1: Effects of PDTC on LPS-induced NO production and related gene expression in RAW 264.7 cells.

## Abbreviations

FLR	Fermented lotus root
LA	Linoleic acid
LPS	Lipopolysaccharide
TLR4	Toll-like receptor 4
NF-κB	Nuclear factor-kappa B
IκBα	Inhibitor of kappa B
MAPK	Mitogen-activated protein kinase
NO	Nitric oxide
iNOS	Inducible nitric oxide synthase
COX-2	Cyclooxygenase-2
TNF-α	Tumor necrosis factor-alpha
IL-1β	Interleukin-1 beta
IL-6	Interleukin-6
*Nos2*	Nitric oxide synthase 2
*Ptgs2*	Prostaglandin-endoperoxide synthase 2
ERK	Extracellular signal-regulated kinase
JNK	c-Jun N-terminal kinase
GC-FID	Gas chromatography with flame ionization detector
VC	Vehicle control
CCK-8	Cell counting kit-8
DAPI	4′,6-Diamidino-2-phenylindole dihydrochloride
ANOVA	Analysis of variance
SEM	Standard error of the mean
