# Supplementary material for: Anti-Inflammatory Effects of Fermented Lotus Root and Linoleic Acid in Lipopolysaccharide-Induced RAW 264.7 Cells
Source: Life (Basel). 2020 Nov 19;10(11):293. doi: 10.3390/life10110293 (PMC7699317; doi:10.3390/life10110293)
Supplement: Supplementary file 1 [file life-10-00293-s001.pdf]

life

Supplementary Materials

Anti-Inflammatory Effects of Fermented Lotus Root and Linoleic Acid in Lipopolysaccharide-Induced RAW 264.7 Cells

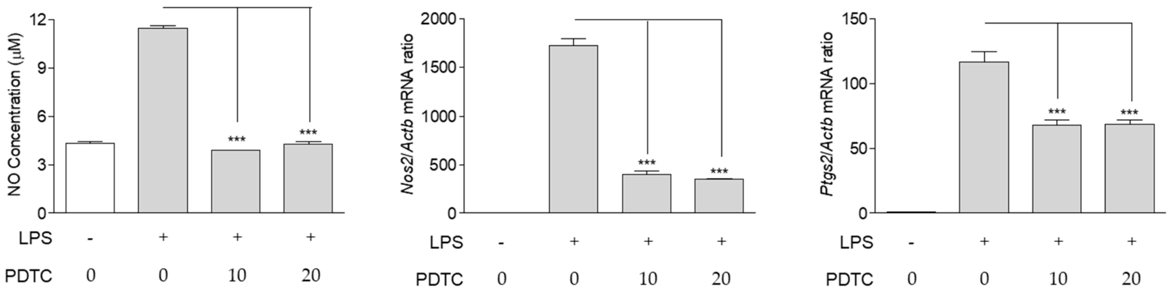

(a) (b) (c)

Figure 1. Effects of PDTC on LPS-induced NO production and related gene expression in RAW 264.7 cells. RAW 264.7 cells were co-treated with LPS (1 μg/mL) and PDTC (10 or 20 μM) for 24 h. (**a**) NO production in culture medium. mRNA expression of (**b**) *Nos2* and (**c**) *Ptgs2* quantified by RT-PCR. The data represent mean ± SEM. ***$p < 0.001$ compared to LPS-only treated group.

Publisher's Note: MDPI stays neutral with regard to jurisdictional claims in published maps and institutional affiliations.

© 2020 by the authors. Submitted for possible open access publication under the terms and conditions of the Creative Commons Attribution (CC BY) license (http://creativecommons.org/licenses/by/4.0/).
